# Hepatitis E Virus Genotype 4, Nanjing, China, 2001–2011

**DOI:** 10.3201/eid1909.130013

**Published:** 2013-09

**Authors:** Xing Dai, Chen Dong, Zhenxian Zhou, Jiuhong Liang, Min Dong, Yan Yang, Jianguang Fu, Hua Tian, Song Wang, Jie Fan, Jihong Meng, Michael A. Purdy

**Affiliations:** Southeast University School of Medicine, Nanjing, China (X. Dai, C. Dong, Z. Zhou, J. Liang, M. Dong, Y. Yang, J. Fu, H. Tian, S. Wang, J. Fan, J. Meng);; Nanjing Second Hospital, Nanjing (Z. Zhou);; Centers for Disease Control and Prevention, Atlanta, Georgia, USA (M.A. Purdy)

**Keywords:** hepatitis E virus, viruses, hepatitis E, acute infection, evolution, genotype 4, Nanjing, China

## Abstract

During 2001–2011, hepatitis E virus (HEV) was found in the blood of patients in Nanjing, China. All HEV-positive patients had virus genotype 4; subgenotype 4a was predominant. The effective population of HEV in Nanjing increased in ≈1980 and continued until ≈2003 when it plateaued.

Hepatitis E virus (HEV) is a positive-sense, single-stranded RNA virus of the family *Hepeviridae*. Strains of HEV infecting swine, boars, deer, mongooses, rabbits, and humans belong to the genus *Hepevirus* (*1*). Hepeviruses are divided into 4 genotypes that cause sporadic and epidemic outbreaks of hepatitis E in humans. Epidemic outbreaks occur primarily in developing countries in Asia and Africa, and sporadic outbreaks occur worldwide (*2*,*3*). A study showed that HEV causes autochthonous hepatitis E in industrialized countries (*4*).

Genotypes 1 and 2 are transmitted from human to human and cause epidemic and sporadic outbreaks (*3*,*5*). Genotypes 3 and 4 are transmitted to humans zoonotically (*6*) and cause primarily sporadic cases of hepatitis E. The dominant genotype of HEV in China is genotype 4 (*5*,*7*,*8*). The purpose of this study was to analyze the prevalence and genotypes of HEV among infected patients in Nanjing, China.

## The Study

A total of 15,910 patients admitted to the Second Hospital in Nanjing, Jiangsu Province, China, because of suspected acute viral hepatitis during January 1, 2001–April 30, 2011, were evaluated by physicians to determine whether they were infected with HEV. Informed consent was obtained from all patients participating in this study. This study was conducted in accordance with national ethics regulation and was approved by the research ethics committee of Southeast University in Nanjing. Because several physicians evaluated the patients, a variety of clinical symptoms were used to diagnose viral hepatitis. Patients were tested for HEV by using serologic analysis. A total of 813 patients who were positive for IgM against HEV were tested for HEV RNA.

ELISAs for antibodies against HEV (Wan Tai Biologic Pharmacy Enterprise Co. Ltd, Beijing, China) were performed according to the manufacturer’s instructions. In-house sandwich enzyme immunoassays were used according to Dong et al. (*9*).

HEV primers were designed to amplify a 691-bp segment from open reading frame 2 genotypes 1–4 (Table 1). RNA extraction and cDNA sequencing were performed as described (*10*). Sequences identified have been deposited in GenBank under accession nos. JX997438–JX997647).

**Table 1:** Primers used for RNA extraction and cDNA synthesis of hepatitis E, virus, Nanjing, China*

Primer	Sequence, 5′→3′	Type
JM2	CCG ACA GAA TTG ATT TCG TCG GC	EF
JM36	CAT YTT AAG RCG CTG MAG CTC AGC	ER
JM3	TYG TCT CRG CCA ATG GCG AGC	IF
JM35	CGR CAY TCM GGG CAR AAR TCA TC	IR
*E, external; F, forward; R, reverse; I, internal.		

A total of 210 sequences were aligned by using ClustalX (*11*) and trimmed at their termini to remove gaps by using BioEdit version 7.0.5.3 (*12*). All sequences (n = 178) without a collection date were removed from the dataset. This sequence set was analyzed to estimate the time to the most recent common ancestor and to generate a skyline plot. These sequences align with open reading frame 2 sequence at nt 6454–6966 (GenBank accession no. AJ272108).

**Figure 1:**
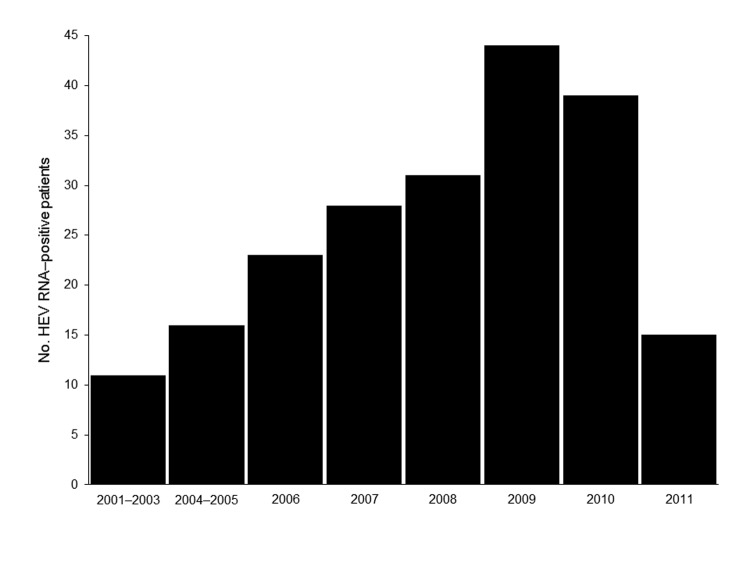
Hepatitis E virus (HEV) RNA–positive patients at Affiliated Second Hospital, Nanjing, China. Data for 2011 are for the first 4 months of the year only.

Sequencing confirmed that all 210 HEV RNA–positive patients were infected with HEV genotype 4. This exclusivity probably reflects the recent trend from infection by genotype 1 toward infection by genotype 4, and the dominance of genotype 4 in Nanjing is similar to that in other regions of China (*7*–*9*,*13*). Although the year of collection for serum specimens was recorded during 2006–2011, before 2006, specific years of collection were not recorded. Records show there were 11 HEV RNA–positive patients at the hospital during 2001–2003 and 16 HEV RNA–patients during 2004–2005. During 2006–2010, the increase in the number of HEV RNA–positive patients was nearly linear. The total number of acute hepatitis E cases during 2011 could not be determined because patients were examined only through the end of April 2011 (Figure 1).

Subgenotypes were determined by creating a neighbor-joining phylogenetic tree from all patient sequences and reference sequences obtained from GenBank: EF077630 for subgenotype 4a, EU676172 for 4b, AB193177 for 4c, GU361892 for 4d, AY723745 for 4e, AB220974 for 4f, and AB108537 for 4g by using ClustalX (*11*). Subgenotypes detected were 4a, 4b, 4c, 4d, 4g, and 3 sequences that appear to belong to an undescribed subgenotype and are most similar to GenBank sequences AB369690, AB521805, AB521806, DQ450072, and EF570133. The predominant subgenotype was 4a (50.2%) (Table 2). Subgenotypes 4b, 4c, 4d, and 4g had prevalences of 5.4%, 9.3%, 32.7% and 1.0%, respectively. The unknown subgenotype had a prevalence of 1.5%.

**Table 2:** Distribution of hepatitis E virus subgenotypes, Nanjing, China 2001–2011

Subgenotype	2001–2003	2004–2005	2006	2007	2008	2009	2010	2011*	No. (%)	Mean ± SD
4a	6	11	11	11	16	24	17	6	103 (50.2)	12.9 ± 6.0
4b	0	0	2	1	1	4	2	1	11 (5.4)	1.4 ± 1.3
4c	2	1	0	4	6	1	4	1	19 (9.3)	2.4 ± 2.1
4d	3	4	9	9	8	12	16	6	67 (32.7)	8.4 ± 4.2
4g	0	0	0	1	1	0	0	0	2 (1.0)	0.3 ± 0.5
4?†	0	0	0	1	0	1	0	1	3 (1.5)	0.4 ± 0.5

*Sequences from 2011 are for the first 4 months of the year. †Putative new subgenotype.

To examine whether there was a change in the prevalence in any of the 3 most abundant subgenotypes (4a, 4c, and 4d) detected, we conducted χ^2^ analyses. Numbers of subgenotype sequences found for each subgenotype were compared with every other subgenotype in adjacent time intervals. In addition, numbers of subgenotypes were compared between the first time period (2001–2003) and 2010 and 2011. The p values for all comparisons were >0.11 except for comparison between subgenotypes 4a and 4c from 2008 with those from 2009 (p = 0.04). Values for subgenotypes 4b, 4g, and the unknown subgenotype were all within 2 SD of their respective means (Table 2). This finding indicates that there were no major differences in subgenotype prevalence over time.

**Figure 2:**
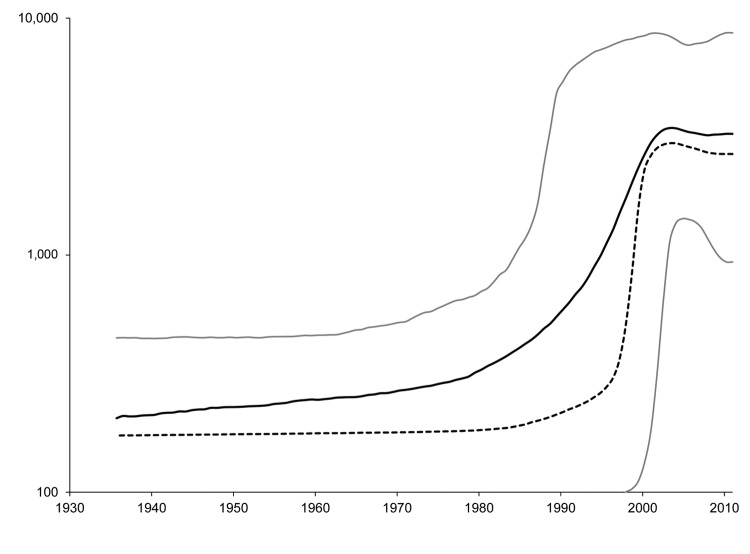
Effective populations of hepatitis E virus, Nanjing, China, calculated from sequences with known years of collection. Solid black line, mean effective population; dashed black line, median effective population; gray lines, the upper and lower limits of the 95% highest posterior density. N_e_ is the effective population with HEV, and τ is the generation time of the virus in years.

BEAST version 1.71 (*14*) was used to estimate the time to the most recent common ancestor and to create a skyline plot as described (*13*) but with use of the Kimura 3-parameter substitution model. Calculation of the time to the most recent common ancestor for sequences with confirmed dates of collection estimated that the most recent common ancestor for these sequences existed 102.5 years ago (range 69.1–141.4 years ago). A skyline plot created from the dated sequences suggests that the effective population of HEV in Nanjing increased slightly more than 1 log. This increase started in ≈1980 and continued until ≈2003, when the effective population of HEV genotype 4 in Nanjing reached a plateau (Figure 2).

## Conclusions

The current findings are different from those reported by Purdy and Khudyakov (*13*), which showed an increase in the effective population of HEV genotype 4 in China from ≈1900 through ≈1940 when the effective population plateaued. Although both plots show an increase in the effective population that plateaus, the periods for these changes differed.

There are 3 potential causes for these differences. First, because the earlier plot (*13*) was constructed with fewer sequences, sampling error may be a factor. Second, the earlier plot nonetheless used longer sequences with many more segregating sites. Third, the earlier plot included sequences originating from a larger geographic area. The second and third reasons could potentially result in more sites with substitutions, resulting in higher sequence diversity, which could extend time estimates further into the past. Because of these 3 disparate factors, we cannot ascertain which plot would be more accurate or whether both plots are correct within their specific contexts. Nevertheless, the common feature of both plots is that the effective population of HEV genotype 4 has increased and subsequently reached a plateau.

These data suggest that HEV genotype 4 has undergone a relatively recent expansion in the late twentieth century in Nanjing. The most dominant subgenotype is 4a, and subgenotype distribution has not changed over the past decade. Therefore, a stable population of genotype 4 HEV has been established in the Nanjing region of China. This finding is consistent with the trend observed in other areas within China in which decreases in the prevalence of HEV genotype 1 have been accompanied by increases in the prevalence of genotype 4 (*5*,*7*,*8*). Whether this replacement of genotype 1 by genotype 4 reflects changes in the environment, changes in mode of transmission, or introduction of HEV genotype 4 to animal reservoirs to which humans have been exposed requires further studies. Such investigations are particularly urgent given the continuing increase in China of the prevalence of acute hepatitis E associated with HEV genotype 4 (*15*).
